# Association between delta anion gap and hospital mortality for patients in cardiothoracic surgery recovery unit: a retrospective cohort study

**DOI:** 10.1186/s12893-022-01625-9

**Published:** 2022-05-14

**Authors:** Kai Xie, Chao Zheng, Gao-Ming Wang, Yi-Fei Diao, Chao Luo, Ellen Wang, Li-Wen Hu, Zhi-Jian Ren, Jing Luo, Bin-Hui Ren, Yi Shen

**Affiliations:** 1 Department of Cardiothoracic Surgery, Jinling Hospital, School of Clinical Medicine, Nanjing Medical University, Nanjing, 210000 China; 2 Department of Cardiothoracic Surgery, Jinling Hospital, School of Medicine, Southeast University, Nanjing, China; 3 Department of Thoracic Surgery, Xuzhou Central Hospital, Xuzhou Clinical School of Xuzhou Medical College, Xuzhou, China; 4 Department of Cardiothoracic Surgery, Jinling Hospital, Medical School of Nanjing University, Nanjing, China; 5 Department of Cardiothoracic Surgery, Jinling Hospital, Southern Medical University, Guangzhou, China; 6 McMaster University, Hamilton, Canada; 7 Jiangsu Key Laboratory of Molecular and Translational Cancer Research, Department of Thoracic Surgery, Jiangsu Cancer Hospital, Jiangsu Institute of Cancer Research, The Affiliated Cancer Hospital of Nanjing Medical University, Nanjing, China

**Keywords:** Anion gap, Cardiothoracic surgery, Mortality, Acid-basic disturbances, MIMIC III

## Abstract

**Backgrounds:**

High level of anion gap (AG) was associated with organic acidosis. This study aimed to explore the relationship between delta AG (ΔAG = AG_max_ − AG_min_) during first 3 days after intensive care unit (ICU) admission and hospital mortality for patients admitted in the cardiothoracic surgery recovery unit (CSRU).

**Methods:**

In this retrospective cohort study, we identified patients from the open access database called Multiparameter Intelligent Monitoring in Intensive Care III (MIMIC III). A logistic regression model was established to predict hospital mortality by adjusting confounding factors using a stepwise backward elimination method. We conducted receiver operating characteristic (ROC) curves to compare the diagnostic performance of acid–base variables. Cox regression model and Kaplan Meier curve were applied to predict patients’ 90-day overall survival (OS).

**Results:**

A total of 2,860 patients were identified. ΔAG was an independent predictive factor of hospital mortality (OR = 1.24 per 1 mEq/L increase, 95% CI: 1.11–1.39, p < 0.001). The area under curve (AUC) values of ΔAG suggested a good diagnostic accuracy (AUC = 0.769). We established the following formula to estimate patients’ hospital mortality: Logit(P) = − 15.69 + 0.21ΔAG + 0.13age-0.21BE + 2.69AKF. After calculating Youden index, patients with ΔAG ≥ 7 was considered at high risk (OR = 4.23, 95% CI: 1.22–14.63, p = 0.023). Kaplan Meier curve demonstrated that patients with ΔAG ≥ 7 had a poorer 90-day OS (Adjusted HR = 3.20, 95% CI: 1.81–5.65, p < 0.001).

**Conclusion:**

ΔAG is a prognostic factor of hospital mortality and 90-day OS. More prospective studies are needed to verify and update our findings.

**Supplementary Information:**

The online version contains supplementary material available at 10.1186/s12893-022-01625-9.

## Strengths and limitations of this study

1. The study had a large sample size based on the MIMIC III database.
2. ΔAG is associated with hospital mortality and 90-day mortality.
3. There is an inevitable bias in the retrospective observational cohort study design.
4. A large number of patients were excluded due to unavailability of AG data.

## Introduction

Acid-basic disturbances are common in patients who are critically ill. Some methods [1, 2] were exploited to evaluate the acid-basic status. Lactate, pH, bicarbonate, AG, strong ion gap (SIG), etc., are widely applied in clinical practice. The AG is calculated from the difference in serum cation and anion concentrations through the formula: AG = [Na^+^]–[Cl^−^]–[HCO3^−^] [3]. As a simple way to evaluate acid-basic status, AG played an essential role in the diagnosis and prognosis of common critical illness, such as acute kidney injury (AKI) [4], sepsis [5], acute myocardial infarction[6]. It was reported that increased AG might result in elevated intensive care unit (ICU) admission and mortality for emergent patients[7]. While several studies focusing on the relationship between anion gap and mortality in critically ill patients showed different results [8–10].

Meanwhile, increased delta AG (ΔAG) was regarded as related to metabolic acidosis [11], which might be devoted to all-cause mortality. Many relevant studies had been carried out to explore the association. Gabow et al. [12] proposed the concept of ΔAG in 1980 for the first time. ΔAG was reported as a novel predictor of outcome in severe patients [13]. Meanwhile, ΔAG was reported as a predictor of all-cause mortality in the critically ill in the study of Lipnick and his colleagues [14]. A systematic review and meta-analysis [15], including 19 studies, reported that using a single AG measurement for risk stratification in critically ill patients could not be recommended. Thus, whether the ΔAG could predict the mortality of severe patients remains still controversial. To explore the strength of this association, we identified patients admitted to CSRU from the MIMIC III database. Unlike other researchers, we defined ΔAG as the difference between maximum and minimum values of AG during the first 3 days of ICU stay due to the lack of pre-hospital AG data [Formula: ΔAG = AG_max_ − AG_min_].

## Materials and methods

### Data source

This retrospective cohort study was based on the Multiparameter Intelligent Monitoring in Intensive Care III (MIMIC III) database [16] (https://mimic.physionet.org/), which is openly available. The MIMIC III database includes more than 40,000 ICU patients and more than 60,000 ICU admissions in Beth Israel Deaconess Medical Center (Boston, MA, USA) from 2001 to 2012. To apply for access to the database, we completed the National Institutes of Health’s web-based course and passed the Protecting Human Research Participants exam (ID 9006491).

### Data extraction

Structured Query Language (SQL) with PostgreSQL (version 11.5) was applied to extract data [17]. Clinical variables, including demographic characteristics, International Classification of Diseases (ICD-9) codes, physiological findings, commonalities, laboratory tests, scoring systems, and other variables, were extracted from the MIMIC III database. The physiological findings included temperature, respiratory rate, heart rate, mean blood pressure, and SpO2. The comorbidities included coronary heart disease (CHD), hypertension, chronic obstructive pulmonary disease (COPD), diabetes. The laboratory tests included red blood cell (RBC), white blood cell (WBC), platelet, Serum creatinine, blood urea nitrogen (BUN), AG, glucose, albumin, pH, base excess (BE), and lactate. Scoring systems included the sequential organ failure assessment (SOFA) score [18], simplified acute physiology score II (SAPSII) [19], and systemic inflammatory response syndrome (SIRS) score [20]. Postoperative complications including acidosis (ICD-9 code: 2762), acute kindney failure (AKF, ICD-9 codes: 5845, 5846, 5847, 5848, 5849), Arrhythmia (atrial fibrillation: ICD-9 code: 42731; ventricular fibrillation: ICD-9 code: 42741), acute respiratory failure (ICD-9 codes: 51851, 51853, 51881), pneumonia (ICD-9 codes: 99731, 99732), pulmonary embolism (ICD-9 codes: 41511, 41512, 41519) were extracted to conduct subgroup analyses. We also extracted variables including mechanical ventilation (ICD-9 codes: 9390, 9670, 9671, 9672), ICU length of stay (LOS) and hospital LOS. Survival information from Social Security Death Index records were extracted, and the OS was calculated for the dead patients.

### Definition of AKF

AKF was defined according to the Kidney Disease: Improving Global Outcomes (KDIGO) criteria [21] as an absolute increase of serum creatinine of > 0.3 mg/dL within 48 h or a relative increase of > 50% in no more than 7 days.

The selection criteria were as follows: (1) Adults patients (age ≥ 18 years); (2) patients admitted in CSRU; (3) Only first ICU admission was eligible; (4) ICU LOS > 1. The exclusion criteria were: (1) without AG data in ICU admission; (2) individual data missing > 5%; (3) patients who died during ICU stay.

The exposures of interest were ΔAG. The primary outcome of interest was hospital mortality, defined as death during the hospital stays. The secondary endpoints included 90-day mortality and overall survival.

### Patient and public involvement

Patients and/or the public were not involved in the design, or conduct, orreporting, or dissemination plans of this research.

### Statistical analysis

Continuous variables were presented in the tables as the mean with standard deviation or median with interquartile ranges (IQR). The Student T-test, or Wilcoxon rank-sum test was applied to compare the difference between groups as appropriate. Categorical variables were presented as a percentage, and the X^2^ test or Fisher exact test was conducted to compare the difference between groups. The missing data were filled by multiple compensations. To explore the crude relationship between ΔAG and hospital mortality, the Lowess Smoothing technique was performed. A stepwise backward elimination method with a significance level of 0.05 was applied to establish the logistic regression models, which used ΔAG as a design variable. A variance inflation factor (VIF) was utilized to estimate the potential multicollinearity, with a value of ≥ 5 indicating multicollinearity. The goodness of fit was assessed for the logistic regression models. Based on the presence or absence of postoperative complications, subgroup analyses were conducted. Sensitivity analysis comparing baseline characteristics between patients with/without AG data was conducted. ROC curves were depicted to show the diagnostic performance, and the appropriate cut-off values were determined by calculating the Youden index (sensitivity + specificity − 1). Cox regression model and Kaplan Meier curve were utilized to identify prognostic factors of 90-day OS. Stata/SE 16.0 (Stata Corp LLC, college station, USA) and R software (version 4.0.0) were applied in performing all statistical analyses. Statistical significance was defined as a two-tailed p < 0.05.

## Results

### Population and baseline characteristics

**Fig. 1:**
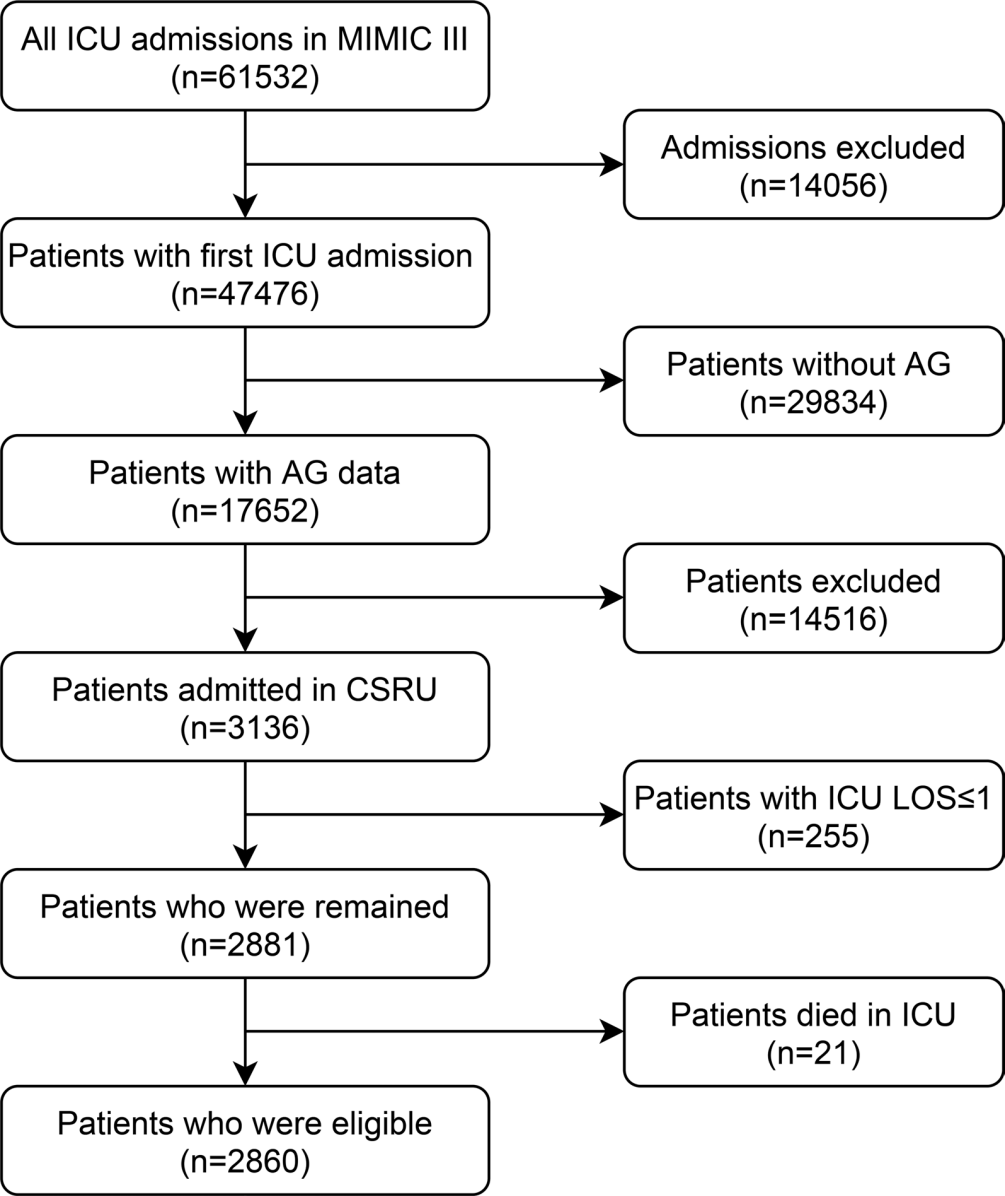
Selecting flow diagram

A total of 2860 patients were identified from the MIMIC III database. The selection flow diagram is presented in Fig. 1. Reasons for CSRU admission are presented in Table 1. Generally, 2261 (79.1%) patients are white. The average age is 67.8 ± 12.1 years, and 1934 (67.6%) patients are male. The median follow-up period was 277 days. The median duration between maximum AG and minimum AG measurements was 0.96 days (IQR: 0.42–2.00). Comparison of characteristics between survivors and non-survivors are presented in Table 2. There were 2841 survivors and 19 deaths, establishing an initial hospital mortality rate of 0.7%. ΔAG of non-survivors is significantly higher than survivors (median: 7 vs. 2, p < 0.001). The incidences of AKF (57.9% vs. 13.4%, p < 0.001) and acidosis (21.1% vs. 5.1%, p = 0.015) are significantly higher in death group.

**Table 1:** Reason for CSRU admission

Reasons for admission	n (%)
Coronary artery disease, n (%)	1005 (35.1)
Aortic valve disease, n (%)	338(11.8)
Mitral valve disease, n (%)	278 (9.7)
Chest pain, n (%)	252 (8.8)
Aortic aneurysm, n (%)	145 (5.1)
Infection or inflammation, n (%)	83 (2.9)
Aortic dissection, n (%)	80 (2.8)
Myocardial infarction, n (%)	80 (2.8)
Congestive heart failure, n (%)	76 (2.7)
Angina, n (%)	73 (2.6)
Chest mass, n (%)	53 (1.9)
Acute coronary syndrome, n (%)	38 (1.3)
Dyspnea, n (%)	32 (1.1)
Atrial fibrillation, n (%)	29 (1.0)
Congenital heart disease, n (%)	27 (0.9)
Other diseases, n (%)	271 (9.5)

**Table 2:** Comparisons of characteristics between Survivors and Non–survivors

Variable	Survivors (n = 2841)	Non–survivors (n = 19)	p value
Age (y)	67.7 ± 12.1	81.2 ± 8.6	< 0.001*
Male, n (%)	1927 (67.8)	7 (36.8)	0.002*
White, n (%)	2249 (79.2)	12 (63.2)	0.088
Body mass index, Kg/m^2^	28.9 ± 6.0	28.8 ± 6.3	0.986
Ventilation, n (%)	2428 (85.5)	11 (57.9)	0.001*
SOFA	5(3–7)	6(4–8)	0.109
SAPS II	36.8 ± 12.5	51.2 ± 13.5	< 0.001*
SIRS	2.8 ± 0.9	3.2 ± 0.9	0.077
Comorbidities			
COPD, n (%)	20 (0.7)	0 (0)	> 0.999
Hypertension, n (%)	1780 (62.7)	6 (31.6)	0.005*
Diabetes, n (%)	910 (32.0)	7 (36.8)	0.654
AG initial (mEq/L)	11.3 ± 2.6	15.8 ± 4.1	< 0.001*
AG_max_ (mEq/L)	12.9 ± 3.3	21.9 ± 8.2	< 0.001*
ΔAG (mEq/L)	2 (1–5)	7 (4–15)	< 0.001*
ΔNa + (mEq/L)	7 (5–9)	13 (5–20)	0.012*
ΔCl − (mEq/L)	4 (3–7)	4 (0–9)	< 0.001*
ΔHCO3 − (mEq/L)	7 (5–9)	14 (7–19)	< 0.001*
Postoperative complications, n (%)			
AKF	381 (13.4)	11 (57.9)	< 0.001*
Acidosis	146 (5.1)	4 (21.1)	0.015*
Arrhythmia	1175 (41.4)	13 (68.4)	0.017*
Acute respiratory failure	151 (5.3)	5 (26.3)	0.003*
Pneumonia	26 (0.9)	2 (10.5)	0.014*
Pulmonary embolism	21 (0.7)	0 (0.0)	> 0.999
Laboratory indexes on POD1			
Serum creatinine (mg/dL)	0.9 (0.8–1.2)	1.5 (0.8–2.2)	0.001*
Urea nitrogen (mg/dL)	19 (14–26)	38 (22–63)	< 0.001*
Glucose (mg/dL)	127.6 ± 18.9	144.2 ± 51.8	< 0.001*
pH	7.40 ± 0.06	7.31 ± 0.13	< 0.001*
Base excess (mEq/L)	1 (0–3)	− 1 (− 9–1)	< 0.001*
Lactate (mmol/L)	1.3 (1–1.6)	1.7 (1.4–2.8)	0.002*

*SOFA* sequential organ failure assessment, *SAPSII* simplified acute physiology score II, *SIRS* systemic inflammatory response syndrome, *AKF* acute kidney failure, *COPD* chronic obstructive pulmonary disease, *AG* anion gap

Considering that a large proportion of patients in CSRU (4034/6894) were excluded due to the unavailability of AG data, we furtherly conducted a sensitivity analysis, comparing the basic characteristics among patients with/without AG data (Table 3). It demonstrated that the two groups of patients were comparable without significant difference observed.

**Table 3:** Comparison of characteristics among patients with/without AG data

Variables	Patients with AG (N = 2860)	Patients without AG (N = 4034)	p value
Age (y)	67.9 ± 12.1	67.8 ± 12.0	0.734
Male, n (%)	1934 (67.6)	2694 (66.8)	0.464
White, n (%)	2261 (79.1)	3184 (78.9)	0.899
Body mass index, Kg/m^2^	28.8 ± 6.0	28.7 ± 5.7	0.483
SOFA	5 (3–7)	5 (3–6)	0.128
SAPSII	36.9 ± 12.5	36.4 ± 11.7	0.089
SIRS	3 (2–4)	3 (2–4)	0.532
Operation types, n (%)			
CABG	1283 (44.9)	1816 (43.8)	0.302
Valve replacement or valvoplasty	621 (21.8)	958 (23.1)	
Resection of vessls	232 (8.1)	369 (8.9)	
Others	724 (25.2)	1004 (24.2)	
Postoperative complications, n (%)			
AKF	392 (13.7)	544 (13.5)	0.792
Acidosis	150 (5.2)	206 (5.1)	0.798
Arrhythmia	1188 (41.5)	1692 (41.9)	0.737
Acute respiratory failure	156 (5.5)	230 (5.7)	0.660
Pneumonia	28 (1.0)	48 (1.2)	0.409
Pulmonary embolism	20 (0.7)	36 (0.9)	0.379
Hospital mortality	19 (0.7)	35 (0.9)	0.295
ICU LOS, median (IQR), day	2.1 (1.3–3.4)	2.3 (1.3–3.6)	0.122
Hospital LOS, median (IQR), day	7.6 (5.8–11.3)	7.9 (5.9–11.5)	0.145

*SOFA* sequential organ failure assessment, *SAPSII* simplified acute physiology score II, *SIRS* systemic inflammatory response syndrome, *CABG* coronary artery bypass graft, *AKF* acute kidney failure, *ICU* intensive care unit, *LOS* length of stay, *IQR* interquartile ranges

### Primary outcomes

**Fig. 2:**
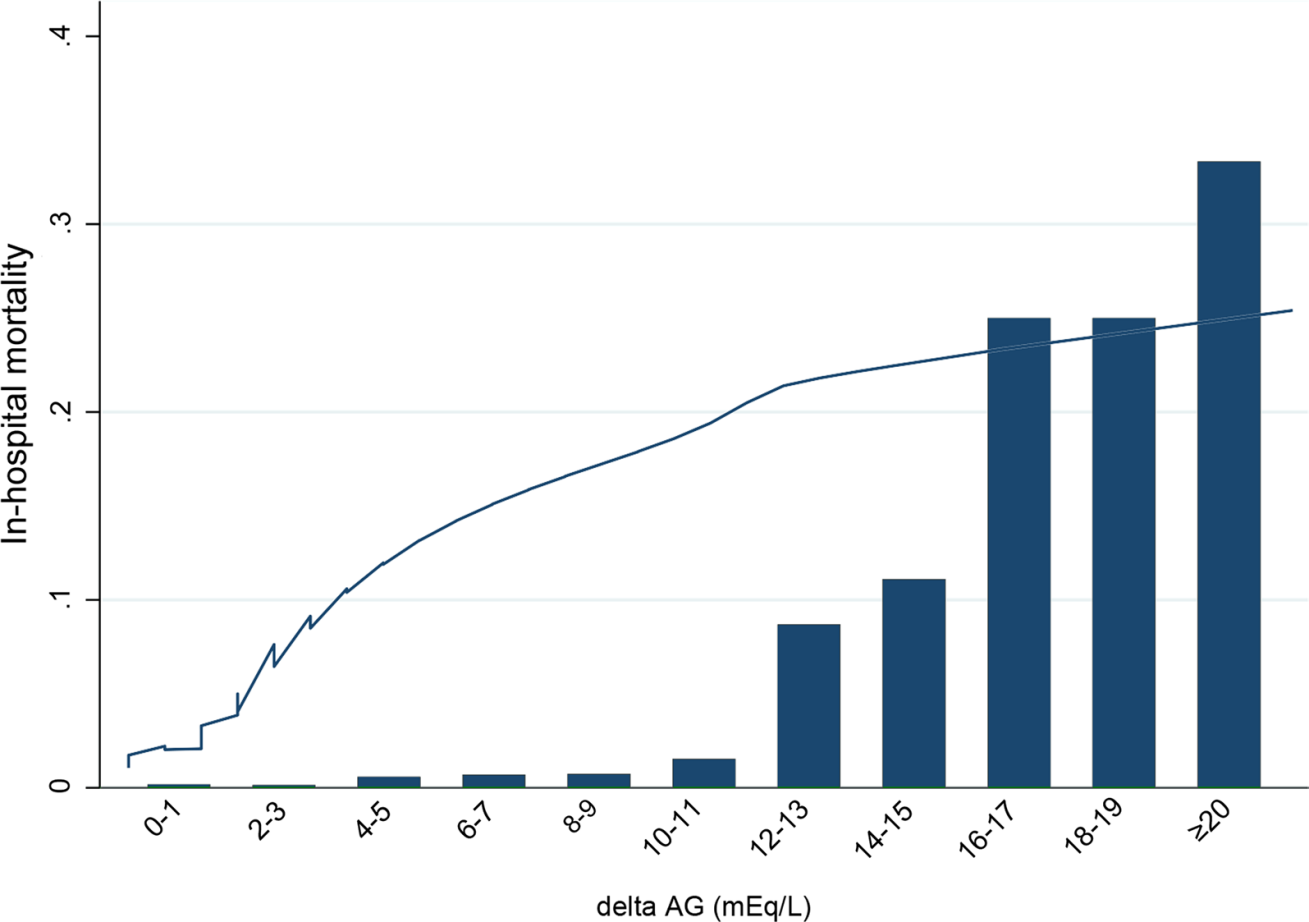
The crude association between ΔAG and hospital mortality using Lowess smoothing technique

The relationship between ΔAG and hospital mortality was presented in Fig. 2, using the Lowess Smoothing technique. Significantly progressive increase of hospital mortality was observed with the increase of ΔAG (χ^2^ for trend, p < 0.001), especially when ΔAG ≥ 12. To identify the cut-off value of ΔAG, we utilized the ROC curve and calculated the Youden index. It indicated that ΔAG ≥ 7 was the optimal cut-off value (Sensitivity: 57.89%, specificity: 87.40%, AUC = 0.769).

We conducted univariate analyses of variables in **Table ****1** to recognize prognostic factors of hospital mortality (Table 4). Using the stepwise backward elimination method, we established the logistic regression model in which ΔAG, age, gender, race, BMI, mechanical ventilation, SOFA score, SAPSII score, SIRS score, AKF, acidosis, diabetes, serum creatine, urea nitrogen, base excess, and lactate were considered. Besides, interaction term between ΔAG and AKF was forced into the final models. Finally, ΔAG, age, base excess, AKF, and ΔAG × AKF were included in the logistic model (**Model 1**), demonstrating a clear relationship between ΔAG and hospital mortality for patients admitted in CSRU (OR = 1.24 per 1 mEq/L increase, 95% CI: 1.11–1.39, p < 0.001). Besides, we found age (OR = 1.14, 95% CI: 1.07–1.21, p < 0.001), base excess (OR = 0.81, 95% CI: 0.72–0.90, p < 0.001), and AKF (OR = 14.87, 95% CI: 2.81–77.55) were independent prognostic factors of hospital for patients in CSRU. Therefore, we proposed the following formula to estimate hospital mortality for patients who underwent cardiothoracic surgery:

$${\text{Logit}}\left( {\text{P}} \right) = \, - {15}.{69} + 0.{21}\Delta {\text{AG}} + 0.{\text{13age}} - 0.{\text{21BE}} + {2}.{\text{69AKF}},$$

**Table 4:** Univariate and multivariate analysis for ΔAG predicting hospital mortality

Variables	Odds ratios	β	95% confidence interval	p value
Univariate logistic regression				
ΔAG (≥ 7 vs < 7)	9.54	2.26	3.81–23.87	< 0.001
ΔAG, continuous	1.24	0.21	1.16–1.32	< 0.001
Gender (male vs female)	0.28	− 1.28	0.11–0.70	0.007
Race (white vs non-white)	2.21	0.80	0.87–5.65	0.096
Age	1.15	0.14	1.08–1.22	< 0.001
Body mass index	0.99	0.00	0.93–1.08	0.986
Serum Creatinine	1.34	0.29	1.12–1.61	0.002
Urea nitrogen	1.04	0.04	1.03–1.06	< 0.001
Lactate	1.87	0.62	1.55–2.24	< 0.001
Base excess	0.75	− 0.28	0.70–0.82	< 0.001
White blood cell	1.06	0.05	0.99–1.12	0.108
AKF	8.87	2.18	3.55–22.21	< 0.001
Acidosis	4.92	1.59	1.63–15.01	0.005
Diabetes	1.24	0.21	0.49–3.15	0.655
SAPSII	1.07	0.07	1.04–1.10	< 0.001
SOFA	1.17	0.15	1.01–1.35	0.040
SIRS	1.66	0.51	0.94–2.93	0.080
Multivariate logistic regression				
* Model 1*				
ΔAG, continuous	1.24	0.21	1.11–1.39	< 0.001
Age	1.14	0.13	1.07–1.21	< 0.001
Base excess	0.81	− 0.21	0.72–0.90	< 0.001
AKF	14.87	2.69	2.81–77.55	0.001
ΔAG × AKF	0.84	− 0.18	0.70–1.01	0.051
Constant		− 15.69	− 20.98 to − 10.40	< 0.001
* Model 2*				
ΔAG (≥ 7 vs < 7)	4.23	1.44	1.22–14.63	0.023
Age	1.14	0.13	1.07–1.21	< 0.001
Base excess	0.78	− 0.25	0.70–0.86	< 0.001
AKF	7.81	2.06	1.59–38.47	0.011
ΔAG × AKF	0.90	− 0.10	0.75–1.09	0.278

The mean VIF of model 1 was 2.78

*AG* anion gap, *AKF* acute kidney failure, *SAPSII* simplified acute physiology score II, *SOFA* sequential organ failure assessment, *SIRS* systemic inflammatory response syndrome

*Notes* ΔAG: mEq/L; Age: years; BE: base excess, mEq/L; AKF, acute kidney failure, 1 for present and 0 for absent.

Meanwhile, we established another logistic model using ΔAG (≥ 7 vs < 7) as a design variable (**Model 2**). The result indicated that ΔAG, age, base excess, and AKF were still independent prognostic factors of hospital mortality. Compared with ΔAG < 7, patients with ΔAG ≥ 7 had a more than three-fold increased risk of hospital mortality (OR = 4.23, 95% CI: 1.22–14.63, p = 0.023). Significant interaction effect between ΔAG and AKF was not observed in both **model 1** and **model 2** (both p > 0.05).

### Subgroup analysis

Considering that the presence of postoperative complications might influence the results, we conducted a subgroup analysis (Additional file 1: Fig. S1). The results indicated that association between ΔAG and hospital mortality was stable except when patients had arrhythmia and/or pneumonia.

**Fig. 3:**
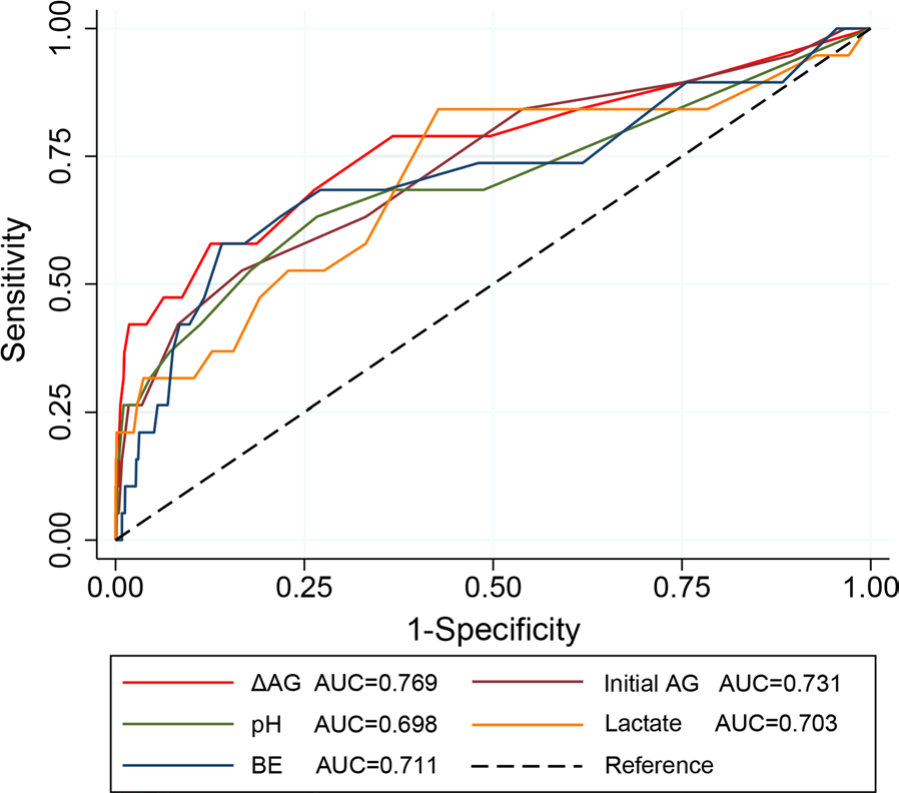
ROC curves of acid–base variables in predicting hospital mortality

We conducted ROC curves to evaluate the diagnostic efficiency of Acid–Base variables, including ΔAG, initial AG, lactate, pH, and BE (Fig. 3). ΔAG demonstrated a good predictive performance in predicting hospital mortality (AUC = 0.769), higher than other acid–base indicators.

### Secondary outcomes

**Fig. 4:**
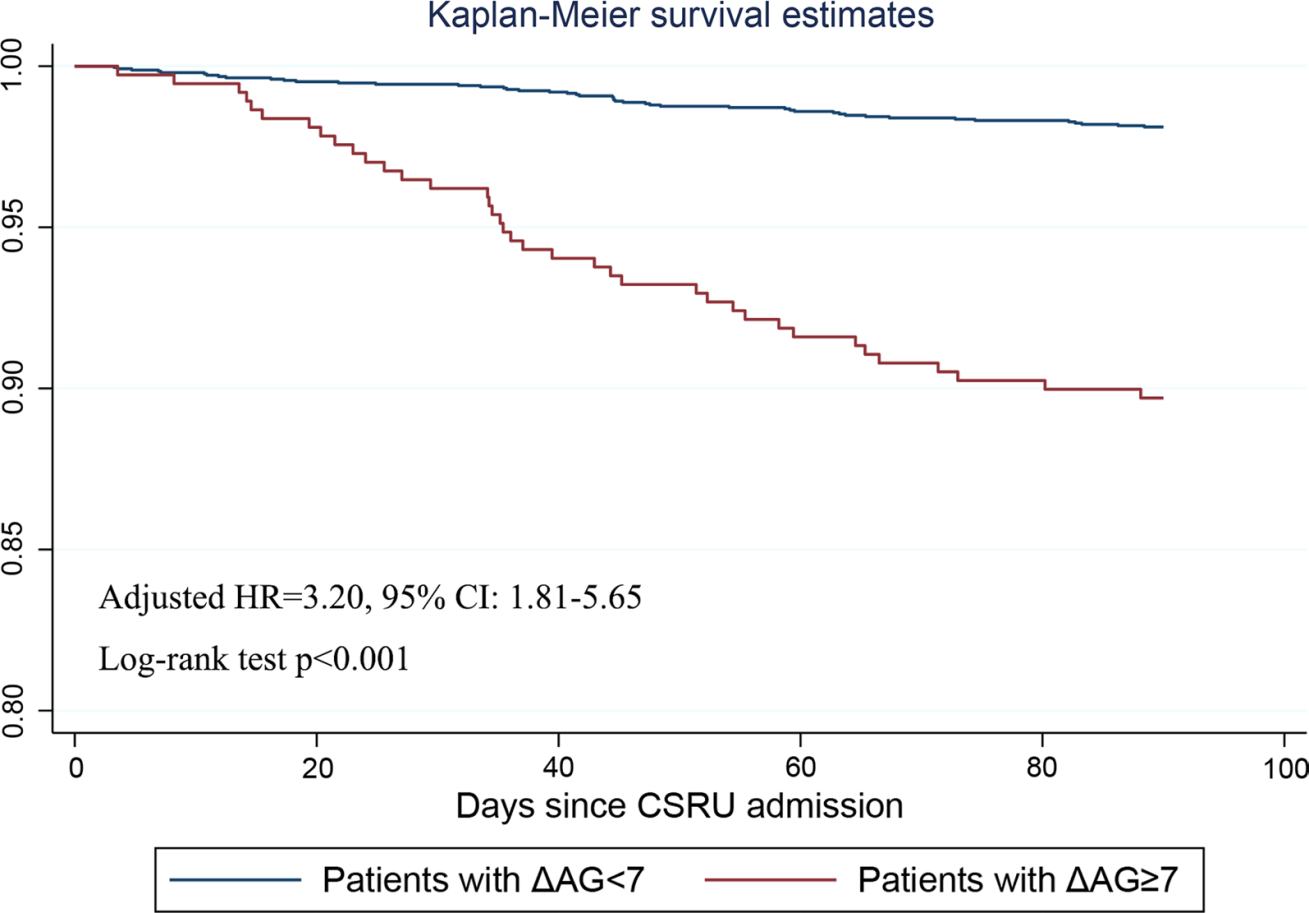
Kaplan Meier curve of 90-day OS stratified by ΔAG ≥ 7 and ΔAG < 7

Patients with ΔAG ≥ 7 had a significantly shorter OS than those with ΔAG < 7 (median OS: 113.7 vs 341.4 days). Considering that all patients were followed up for at least 3 months, we conducted multivariate Cox regression analysis and Kaplan Meier curve to identify prognostic factors of 90-day OS. There are totally 85 patients died within 90 days of admission, including 47 patients in low ΔAG group (ΔAG < 7, n = 2491) and 38 in high ΔAG group (ΔAG ≥ 7, n = 369). A higher 90-day mortality rate was observed in patients with ΔAG ≥ 7 (Adjusted HR = 3.20, 95% CI: 1.81–5.65, p < 0.001) (Fig. 4). Meanwhile, age (HR = 1.05, 95% CI: 1.03–1.08, p < 0.001), and AKF (HR = 4.79, 95% CI: 2.33–9.84, p < 0.001) was found as independent prognostic factors of the 90-day OS. Interaction term between ΔAG and AKF was forced into the Cox regression model but significant effect was not observed (p = 0.583).

## Discussion

The AG was regarded as a crucial indicator in diagnosing acid-basic disturbance, while the association between AG and mortality was unclear. Gabow et al. [12] proposed the concept of ΔAG in 1980. In the study of Lipnick et al. [14], ΔAG was defined as the difference between the initial AG of ICU admission and prehospital admission AG. The ΔAG presented strong predictive efficiency in 30-day all-cause mortality in critically ill patients. Based on the MIMIC III database, Cheng et al. [22] explored the relationship between AG and all-cause mortality in critically ill patients with AKI, suggesting a “U-shaped” relationship between AG and 30-day mortality. In this study, we explored the association between ΔAG and hospital mortality. After adjusting by confounding factors, we found that ΔAG was associated with increased hospital mortality (β = 0.19), and patients with ΔAG ≥ 7 were regarded as at high risk of hospital mortality.

A significant increase of AG (> 30 mEq/L) was regarded as the diagnostic basis of acidosis, including lactic acidosis, ketoacidosis, and acidosis caused by toxins or uremia [12, 23]. For critically ill newborns, AG > 16 mEq/L could predict lactic acidosis [24]. Causes of high AG acidosis include carbon monoxide, cyanide, alcohol intoxication, alcoholic ketoacidosis, toluene, methanol, uremic acidosis, diabetic ketoacidosis, diethylene glycol, paraldehyde, pyroglutamic acid, propylene glycol, iron, ibuprofen, isopropyl alcohol, lactic acidosis, ethylene glycol, and salicylates[3, 25]. A high level of AG was reported to relate to many factors, such as hypertension, reduced cardiopulmonary fitness, and decreased renal function. Accumulation of organic acid might devote to an increased serum AG, which might result in hypertension [26]. Abramowitz and his colleagues reported that a higher serum AG was related to the reduced cardiopulmonary fitness in patients aged 20–49 years [27]. Uremia was one reason for elevated AG, which might cause the death of patients with chronic kidney disease [28]. Besides, elevated anion gap was reported not only to be associated with hyperlactatemia [29] but also a higher mortality of AKF patients [22], which means that lactate and AKF were possible intermediates on a causal pathway between change in anion gap and mortality. Renal failure, diabetic ketoacidosis, and lactic acidosis are the most common causes of high AG acidosis [30]. Graciela et al. reported that critically ill patients may present severe hyperlactatemia with normal values of BE due to that the acidifying effect of severe hyperlactatemia is frequently masked by alkalinizing processes that normalize the BE [31]. Thus, BE might be an important confounding factor. Low cardiac output syndrome often occurs in patients undergoing cardiothoracic surgery due to various reasons such as long operation time, which might cause tissue hypoperfusion and potassium metabolism disorder. ΔAG was a parameter which presented the changes of patients’ pathophysiologic condition during ICU stay. It can be assumed that when the patient’s AG changes greatly, the patient may have the following situations: one is the occurrence of acidosis or the alteration of acid–base balance disorder types, and the other is the higher incidences of postoperative complications such as AKF. Then clinicians would be warned if a patient has a large ΔAG during the ICU stay because they might have a higher mortality according to the present study.

Other biomarkers of metabolic acidosis, such as pH, BE, SIG, and lactate, showed different predictive efficacy in the previous researches [10, 32, 33]. To compare the predictive efficiency of these biomarkers in acid–base balance disorder, the ROC curves were conducted. The calculated AUC values suggested that ΔAG presented a good predictive efficacy, better than initial AG, pH, lactate, and BE, which was similar to Ho’s study [34].

There were several strengths for this study. Primarily, ΔAG-mortality association remains controversial, and most of the previous researches had a small sample size. The study was based on the MIMIC III database, which contained more than 60,000 ICU admissions. In addition, for observational studies, hospital mortality is a fair outcome closely related to clinical practice. Secondary outcomes like 90-day OS was considered too. Moreover, all patients were followed up for at least 90 days after discharge, with a median follow-up period of 230 days.

The limitations of the study are as follows. There is an inevitable bias in the retrospective observational cohort study design. Although confounding factors were included as much as possible in the logistic regression model through a stepwise backward elimination method, other factors like intravenous fluids might influence the AG levels [15]. High ΔAG might be observed during critical state of dying patients which means that postoperative complications and causes of death were important confounding factors. Subgroup analyses according to presence or absence of complications indicated the ΔAG-mortality association a stable one. While, causes of death cannot be provided by MIMIC III database, so we cannot answer the question as to whether ΔAG-mortality association would be affected by the causes of death. The underlying mechanism between higher ΔAG and poor clinical outcomes is still not clear. More researches which are based on causal mediation analysis [35, 36] to investigate the mediating effects of confounding factors on delta AG in terms of mortality are needed in the future studies. Besides, Patients with long operation time were more likely to suffer from acid–base balance disorder after surgery because of tissue ischemia and hypoxia, while these data were not available in the MIMIC III database. Furthermore, only 813 (28.2%) patients had measured the albumin levels, and it was inappropriate to correct for ΔAG using the formula: cAG = AG + (4.4 − albumin) × 2.5 [37, 38]. Besides, the ratio of ΔAG/ΔHCO3^−^ was reported to play a role in diagnosing mixed acid–base imbalance [39, 40], which could be added in future research.

## Conclusion

In aggregate, ΔAG is an independent prognostic factor of hospital mortality and 90-day OS for patients who underwent cardiothoracic surgery. Besides, ΔAG ≥ 7 might be applied as an indicator of risk stratification for severe patients in CSRU.

## Patient and public involvement

Patients and/or the public were not involved in the design, or conduct, or reporting, or dissemination plans of this research.

## Supplementary Information

**Additional file 1. Supplementary Figure 1**. Subgroup analyses according to postoperative complications using forest plots.

## Data Availability

The datasets used and/or analyzed during the current study are available from the corresponding author on reasonable request. However, reanalysis of the full data needs to be approved by MIMIC III Institute.

## Abbreviations

AG: Anion gap
CSRU: Cardiothoracic surgery recovery unit
MIMIC III: Multiparameter intelligent monitoring in intensive care III
ROC: Receiver operating characteristic
OS: Overall survival
AUC: Area under curves
SIG: Strong ion gap
AKI: Acute kidney injury
SQL: Structured query language
CHD: Coronary heart disease
COPD: Chronic obstructive pulmonary disease
RBC: Red blood cell
WBC: White blood cell
BUN: Blood urea nitrogen
BE: Base excess
SIRS: Systemic inflammatory response syndrome
LOS: Length of stay
SOFA: Sequential organ failure assessment
SAPSII: Simplified acute physiology score II
ICU: Intensive care unit
ICD-9: International classification of diseases 9th edition
OR: Odds ratio
HR: Hazard ratio
CI: Confidence interval
