# Supplementary material for: Association between delta anion gap and hospital mortality for patients in cardiothoracic surgery recovery unit: a retrospective cohort study
Source: BMC Surg. 2022 May 14;22:186. doi: 10.1186/s12893-022-01625-9 (PMC9107697; doi:10.1186/s12893-022-01625-9)
Supplement: Supplementary file 1 — Additional file 1. Supplementary Figure 1. Subgroup analyses according to postoperative complications using forest plots. [file 12893_2022_1625_MOESM1_ESM.pdf]

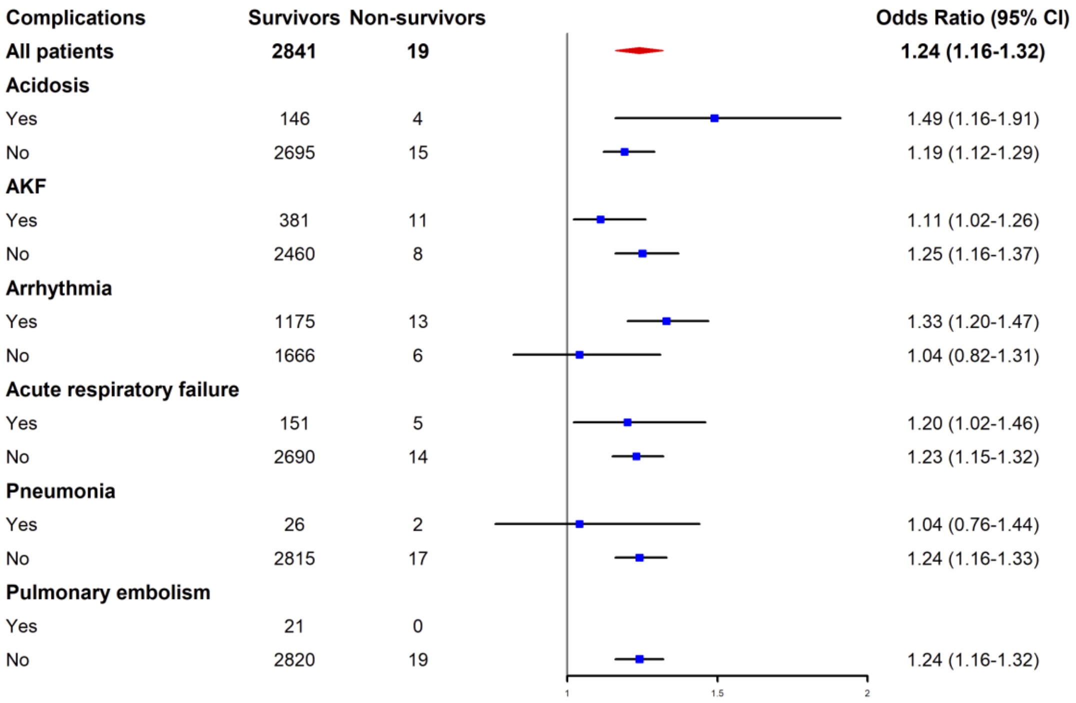

Supplementary Figure 1. Subgroup analyses according to postoperative complications using forest plots
